# The impact of a changed writing environment on students' motivation to write

**DOI:** 10.3389/fpsyg.2023.1212940

**Published:** 2023-10-30

**Authors:** Debra Myhill, Teresa Cremin, Lucy Oliver

**Affiliations:** ^1^School of Education, University of Exeter, Exeter, United Kingdom; ^2^Faculty of Wellbeing, Education and Language Studies, The Open University, Milton Keynes, United Kingdom

**Keywords:** writing, writing motivation, writing environment, community of writers, focus groups

## Abstract

**Introduction:**

The act of writing is widely acknowledged to be a complex and challenging activity, and in parallel, we know that student motivation to write is a predictor of writing performance. So understanding what characteristics of the writing classroom support or foster motivation remains a salient concern. Research has shown that UK teachers are more likely to see themselves as readers than writers, which may affect how they teach writing.

**Methods:**

This paper reports on student focus group interview data from a study which sought to strengthen teachers' sense of themselves as writers, and to examine the impact of this on students' classroom experience of writing and their writing outcomes. The participant teachers experienced a creative writing residential, which established a writing community led by two professional writers, with the goal of changing teachers' professional practice in their own writing classrooms. The study was mixed methods, comprising a randomized controlled trial and a comprehensive qualitative dataset collating data from both the residential and the classroom. This paper presents the qualitative analysis of 32 interviews with 16 student focus groups, exploring their responses to their teachers' changed practices and how it connected with their motivation to write.

**Results:**

The interview analysis shows how many students responded positively to new teaching practices which gave them greater autonomy and choice, and established a more collaborative way of working. This led to increased confidence in and motivation to write.

**Discussion:**

The study highlights the importance of the classroom environment in supporting and sustaining motivation to write, and underlines that motivation is not simply an internal characteristic of an individual but is situated within the context of a community of writers.

## 1. Introduction

The act of writing is well-recognized as cognitively and socially complex: indeed, Flower and Hayes argued that a writer is “*a thinker on a full-time cognitive overload”* (1980, p. 33) and Kellogg (2008) has likened its cognitive demand to that of playing chess. It is a multidimensional construct, requiring mastery of multiple skills, ranging from transcription and orthography, the management of sentence and text structures; the generation of ideas; understanding the expectations of a genre; and navigating the relationship between reader and writer. Perhaps it is not surprising, then, that motivation to write in school can be problematic. Research has suggested that that students' motivation to write appears to decline through schooling (Boscolo and Gelati, 2019; Wright et al., 2020) although, of course, it is also the case that students' general motivation in school declines through adolescence (see, for example, Eccles et al., 1997; Gottfried et al., 2001; Raufelder and Kulakow, 2021). Nonetheless, addressing motivation in writing is important both academically, because so much examination success depends on competence in writing, and socially, because writing is a means of personal communication and expression, and ubiquitous in a digital world. The importance of investigating motivation in writing is further emphasized by studies which show positive links between motivational constructs and writing outcomes (Pajares and Johnson, 1994; Troia et al., 2013; Graham et al., 2017; Camacho et al., 2021). In this article, we investigate writing motivation through a specific focus on the classroom environment for writing, considering student responses to changed teachers' pedagogical practices in teaching writing and how this may connect with their motivation to write. We argue that the nature of the classroom environment is an important factor in nurturing motivation to write.

## 2. Conceptual framework

In line with Wright et al. (2020, p. 153), we define motivation to write as “*the variety of reasons a child may choose to engage in a writing task or decide to take steps to avoid that task.”* This involves the beliefs, values, goals and dispositions that students bring to a writing task (Boscolo and Gelati, 2019), and, crucially, how these are dynamically shaped over time through student experiences of the writing classroom. We adopt an interdisciplinary perspective on writing and writing motivation, in line with Graham (2018) who argues for an integration of cognitive and sociocultural perspectives. In particular, we recognize that the act of writing involves both cognitive mental processes and beliefs and behaviors shaped by classroom and broader social contexts. Accordingly, we have synthesized the literature into four themes which reflect this interdisciplinary perspective in different ways: self-efficacy beliefs about writing; autonomy, choice and control; writing as social practice; and the classroom environment for writing.

### 2.1. Self-efficacy beliefs about writing

The concept of self-efficacy is fundamentally concerned with individuals' personal sense of their capacity to be successful in a task, or as Bandura (1997, p. 3) defined them, they are “*beliefs in one's capabilities to organize and execute the course of action required to produce given attainments.”* These beliefs are powerful because they influence individuals' behavior and affective responses to a task, and the extent to which an individual is willing to engage with a particular task: if we believe we can achieve a task, even though it may be challenging, we are more likely to commit the necessary effort, whereas if we believe we cannot accomplish a task, we are less likely to expend effort. Thus self-efficacy beliefs “*play a central role in the cognitive regulation of motivation*” (Bandura, 1997, p. 122). Given that the act of writing is cognitively demanding, as noted above, even for highly competent writers, it is easy to recognize the inter-relationship between the cognitive demands of writing, students' self-efficacy beliefs about writing and being a writer, and motivation to write. In their study investigating motivation for writing in the middle school years, Wright et al. (2020) make the connection between writing motivation and self-efficacy beliefs, maintaining that “*a student with strong self-beliefs as a writer would be more likely to work hard and persevere through a challenging task, knowing he or she has the necessary skills to be successful”* (p. 162). Similarly, Pajares and Valiante (2006) argue that students' confidence in their capability to write (their self-efficacy beliefs) contributes to their motivation to write and their writing outcomes. This association between self-efficacy beliefs and writing performance has been well-established (for example, McCarthy et al., 1985; Pajares and Johnson, 1994; Pajares and Valiante, 1999). However, Bruning et al. (2013) note that research has tended to view self-efficacy for writing as a unidimensional construct, when different aspects of the act of writing might generate differing self-efficacy beliefs. They posit and test a three-factor model of self-efficacy in writing, addressing *ideation* (generating ideas), *conventions* (mastery of norms of spelling, paragraphing, sentence structure etc), and *self-regulation* (managing decisions and behaviors while writing). Their study found that students did indeed hold different levels of self-efficacy beliefs on these three factors. This included finding stronger relationships between enjoying writing, and self-efficacy for ideation and self-regulation which may “*hint at the possibility of greater affect associated with writers” confidence for thinking of good ideas (ideation) and managing the writing process (self-regulation) than with believing they can capably execute writings' conventions'* (Bruning et al., 2013, p. 35). Given the focus of this article on the classroom environment for writing, and the strong link between self-efficacy and writing motivation discussed here, it seems pertinent to consider whether and which pedagogical practices might support the generation of high self-efficacy beliefs. Summarizing substantive research on this, Pajares and Valiante (2006, p. 167) conclude that meaningful writing activities; greater autonomy; choice in writing assignments; collaborative writing; self-regulation development; instruction well-matched to learning need; less competitive writing environments; and effective modeling practices have all been found to positively support writing self-efficacy beliefs.

### 2.2. Autonomy, choice, and control in writing

One set of influences affecting self-efficacy beliefs for writing noted in Pajares and Valiante's review of self-efficacy and writing (2006), described above, is giving greater autonomy and more choice in writing. Self-determination theory (Ryan and Deci, 2000), one of the major theories of motivation, refers to an individual's sense of whether they are able to make choices and feel in control in a particular domain. One of the core concepts in self-determination theory is autonomy, defined as “*the psychological need to behave according to one's interests and values*” (Turner et al., 2014). Pajares and Valiante (2006) note that control is also a key concept in attribution theory to explain motivation. It seems important, then, to consider the extent to which students in school contexts have autonomy as writers, and whether they feel they do have control and can make their own choices. As students progress through formal schooling, their experience of writing changes, from more typically engaging and expressive writing in the younger phases, through to a widening range of genres and greater emphasis on disciplinary writing. Wright et al. (2019, p. 64) argue that “*by middle school, writing autonomy diminishes as the focus shifts and students are required to produce discipline-specific texts”* and also that their writing experiences offer “*minimal opportunity for creativity and expression.”* Alongside this reduction in choice of *what* to write about, learning about writing inevitably involves explicit teaching about *how* to write, in terms of the genre conventions of different texts and mastering the norms of writing in terms of spelling, punctuation, paragraphing and so on. This can decrease motivation for writing, according to Boscolo and Gelati (2013) because it demands conformity, rather than freedom for self-expression. The study of De Smedt et al. (2020), looking at motivation for reading and writing, found that autonomous motivation decreased with age, and this may correspond to a parallel increase in controlled motivation as the teaching of writing becomes more oriented toward specific outcomes. At the heart of this is a dilemma for teachers: in order to become capable, confident writers, students need to develop proficiency with writing in an increasing range of genres and contexts, but the consequence of this appears to be a demotivating reduction in autonomy and choice.

There are, however, teaching strategies or practices which teachers can adopt which appear to support autonomy. For example, teaching self-regulation skills for writing has been argued to increase student control of the writing process (Hidi and Boscolo, 2006; Pajares and Valiante, 2006; Graham et al., 2017; Wright et al., 2019). In effect, self-regulation shifts control from the teacher to the student, helping them to reflect on and understand how they manage the writing process, and use strategic behaviors to cope with problems or enable writing success. In addition to teaching self-regulation practices, Pajares and Valiante (2006) note that giving greater choice in writing tasks is important for motivation because the increased autonomy generates greater self-efficacy. In England, the experience of school closures during the COVID pandemic has provided unexpected evidence that greater autonomy and choice can affect motivation and enjoyment of writing. The National Literacy Trust's survey (Clarke and Picton, 2020) of 4,140 students in 2020 found that students' enjoyment of writing had increased on previous years, with one in six students reporting that they were writing more during the pandemic lockdowns than previously. Respondents said that lockdown had inspired their writing, given them access to digital formats for writing, and created time and space for thinking and generating ideas. The report authors argue that “*having more time to write freely has contributed to their increased enjoyment of writing. Looking ahead, it seems that providing time for free writing once back in the classroom could help to sustain this positive outcome”* (2020, p. 12). A key point here, however, is not simply about time, but that students were choosing of their own volition to write in this time, reflecting autonomy in their decision-making.

### 2.3. Writing as social practice

Although, in general, motivation research is typically investigated from a psychological perspective, there is also recognition that writing is not only about cognitive processes but also about social and cultural practices. Indeed, the students in Clark and Picton's survey reported writing for social purposes to help them cope with experience of lockdown, and to connect with others. Students learn to write not only through gradual mastery of transcription and composition, but also through situated learning in the “*contexts in which those practices and activities take their functions and meanings”* (Hidi and Boscolo, 2006, p. 152). Contexts for writing are multi-layered, including (national) curriculum contexts, out-of-school and family contexts for writing, and digital contexts for writing. But for many students, the classroom is one of the most powerful contexts for shaping understandings about writing. The writer(s)-within-community model of writing, proposed by Graham (2018), acknowledges the salience of sociocultural perspectives and integrates them with cognitive perspectives. The two core structures of the writers-within-community model are the writing community, representing the social and cultural contexts in which writing occurs, and writers and their collaborators, representing what individuals and groups bring to the act of writing. A writing community is a community of practice (Lave and Wenger, 1991), bringing together people with a shared purpose, and engaging in “*a process of collective learning in a shared domain”* (Wenger-Trayner and Wenger-Trayner, 2015). From a sociocultural perspective, writing communities are characterized by collaboration and learning together, involving discussion and dialogic processes (Moore, 2003; Hidi and Boscolo, 2006; Prior, 2006).

A social practice view positions writing as fundamentally about meaning-making, not just about text production, and frequently advocates authentic writing tasks (see for example, Behizadeh, 2014, and Rodesiler and Kelley, 2017). This connects directly with research on writing motivation where meaningful authentic writing tasks are seen to enhance motivation (Bruning and Horn, 2000; Hidi and Boscolo, 2006). The increase of enjoyment of writing during lockdown, reported in survey Clarke and Picton (2020) may, in part be attributable to the meaningfulness of the self-chosen writing as the students believed that writing during lockdown made them feel better emotionally.

### 2.4. The classroom environment for writing

The notion of writing as social practice occurring within a community of writers points to the significance of considering how the classroom context may shape student motivation for writing. This shifts the focus away from individual characteristics to a more complex, situated perspective (Eccles and Wigfield, 2020; Nolen, 2020) which acknowledges the multiple and laminated systems of meaning constructed in a classroom environment. It creates space for consideration of the motivational climate, defined by Robinson (2023) as the “*characteristics of the educational setting that contribute to shaping motivational beliefs among students in that environment”* (p. 5). Robinson argues that the motivational climate is not simply about observable teaching practices but about how students feel about their teaching and the meanings they create from it. She draws on achievement goal theory which brings together both achievement goals—the mastery or performance goals held by individuals, and goal structures—the teachers' policies and practices in the learning environment and the explicit goal-related messages they convey (Wolters and Taylor, 2012; Bardach et al., 2020). Yet writing research has had surprisingly little to say about how the classroom writing environment might influence student motivation to write, other than frequent references to the technological “environment” for online and digital writing strategies (see Camacho et al., 2021). However, Bruning and Horn (2000) identify four conditions for writing motivation, which draw out the more complex interplay of student, teacher and contextual factors in the classroom:

1. *Nurturing functional beliefs about writing*.
2. *Fostering student engagement through authentic writing goals and contexts*.
3. *Providing a supportive context for writing*.
4. *Creating a positive emotional environment* (p. 27).

Their elaboration of these four conditions includes multiple references which connect well with the earlier discussion of self-efficacy beliefs about writing, autonomy, choice and control, and writing as social practice (for example, positive experiences of writing to boost self-efficacy; writing from personal interest; authentic tasks which connect with real-world experiences; peer feedback; giving students choice of what to write about; a collaborative writing community; and a safe space for writing).

Thus, as this synthesis of the research shows, there is little research, to our knowledge, which addresses the motivational climate of the writing classroom environment. Camacho, Alves and Boscolo (2021) systematic review of research on writing motivation in school concluded that there is a need for future research to give more attention to the relationship between teachers' instructional practices and student motivation, but this assumes, perhaps, a linearity between instructional practices and motivation which belies the situated complexity of the writing environment. Graham's (2018) work on writer(s)-within-community is significant in this respect, positioning learning about writing within a social and cognitive perspective. In this article, we seek to build on this work by investigating what students' responses to a changed classroom environment for writing reveal about its impact on their motivation to write.

## 3. Methodology

The data informing this paper are drawn from focus group interviews with students who were part of a larger study (*Teachers as Writers*). The study set out principally to explore whether a residential writing course, led by professional writers, would change teachers' beliefs about writing and themselves as writers, which would lead to changed teachers' practices in the classroom, and ultimately to improvements in students' writing. In England, teachers of English are more likely to be English Literature graduates (Shortis and Blake, 2010), and more likely to see themselves as readers, rather than writers (Gannon and Davies, 2007). Thus, in classroom practice they are often more expert in teaching reading and literary analysis, than writing. The benefit of teachers being writers themselves is popularly advocated as important in addressing this imbalance, giving teachers both greater confidence as writers and better professional understanding of the writing process: however, robust evidence of this has been limited (Cremin and Oliver, 2017). Moreover, Bruning and Horn (2000, p. 26) maintain that teachers' “*conceptions of writing will provide a model for and shape students' beliefs*” and argue for a strong connection between teachers' beliefs and students' writing motivation.

The study was mixed methods, involving a Randomized Controlled Trial and qualitative data comprising observations of the residential experience and subsequent classroom teaching; interviews with the professional writers, teachers and students involved; and teacher reflective audio-diaries. The project involved 32 teachers from schools in South-West England, teaching classes with students ranging from age 7–14 years old (*n* = 711). There were eight primary (age 7–11) and eight secondary (age 12–14) classes in both the intervention and control group. The participating teachers attended a week-long writing residential at one of Arvon's writing centers (in SW England). The residential focussed on creative writing, and was led by two professional writers. Following the residential, each teacher and a professional writer together planned a narrative writing teaching unit which was then taught in school, including two lessons co-taught by the teacher and writer.

The findings are reported fully elsewhere (Cremin et al., 2020; Myhill et al., 2021), but, in a nutshell, the “teachers as writers” experience impacted teachers' identities as writers, and led to changes in their classroom practice, but it did not lead to an improvement in student writing quality. However, it did have a positive impact on students' motivation and confidence as writers. It is this latter finding which this paper explores, drawing on the qualitative data from student focus group interviews, and addressing the research question—how do students respond to a changed classroom environment for writing, and how does this connect with their motivation to write?

### 3.1. The intervention

**Figure 1:**
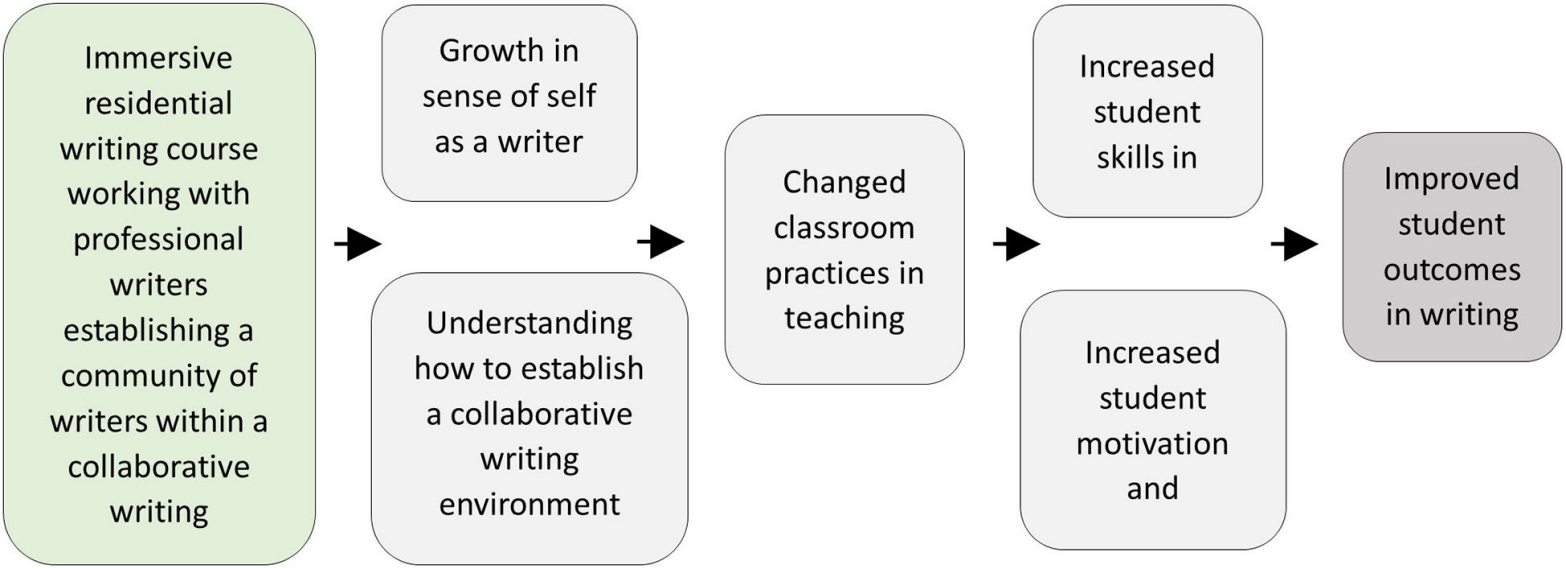
The theory of change model.

Because this paper is primarily concerned with students' perceptions of the changed teaching practices and writing environment following the writing residential, it is important here to consider how the intervention was anticipated to impact on teachers' practices in teaching writing and the writing environment the students would experience. A full overview of the residential programme is provided in Appendix A. In summary, the residential had a daily pattern of writing workshops as a group, one-to-one tutorials with a professional writer, and individual time and space for writing, making use of the natural environment of the residential center. In the workshops, tutors emphasized the recursivity or messiness of the writing process, sharing their own experiences as writers and the value of drawing from personal experience as a source of ideas. They used freewriting repeatedly in the workshop sessions to generate a flow of writing, and used a variety of prompts for writing, such as using artifacts, pictures or personal memories. However, teachers always had freedom to choose what to write about, and what form the writing took—and in the individual time and space for writing, they had autonomy about whether to write, or what pieces of writing to work on. Throughout the residential, the professional writers explicitly emphasized a collaborative writing environment establishing a community of writers, with teachers routinely sharing their writing drafts, and support and feedback from peers actively fostered. The week ended with shared publication of an anthology produced by the teachers and a presentation of writing, intended to give them autonomy, choice and control over what was included or not; to be a final act of collaboration and sharing as a writing community; and overall to boost their sense of self-efficacy as writers. The intention was that this experience would lead to changed classroom practices and ultimately to improved student outcomes in writing. The Theory of Change model is represented in Figure 1.

### 3.2. Data collection and data analysis process

The data drawn on for this paper derive from 16 student focus groups, each interviewed twice. Focus group interviews were chosen because the participants were children, and the group context is less intimidating than individual interviews, and in education, they are viewed as empowering participants, giving primacy to their voices (Bourne and Winstone, 2021). They also allow for participants to respond to and explore the contributions of others, rather than being wholly interviewer-led: “*they involve the interaction of group participants with each other as well as with the researcher-moderator*” (Wilkinson, 2006, p. 223), and thus, they are seen as generating rich, in-depth data, capable of making visible where agreements and disagreements exist (Gill and Baillie, 2018). In planning for and conducting the focus group interviews, we adopted the method recommended by the National Foundation for Education Research (NFER) because of their understanding of researching in classrooms, and because of its acceptability to both schools and researchers in the UK context. They recommended five steps: (1) Develop the questions; (2) Identify the sample; (3) Conduct the interviews; (4) Draw together and analyse the data; and (5) Report the findings (National Foundation for Educational Research, 2013, p. 3).

The first step, therefore, involved designing a semi-structured interview for each of the two interviews. The first interview sought to explore five constructs: their perceptions of writing; their perceptions of the teaching of writing; their understanding of the writing process; their enjoyment of and motivation to write; and their confidence and perceived skills as writers. The second interview was particularly designed to elicit their responses to any changes in how they were taught writing, or changes in their attitudes toward writing (see Appendix B). It allowed us to build on the ideas and responses of the first interview, supporting member-checking through the interviewing process (Harvey, 2015). The interview questions were designed avoiding closed questions, and taking care to frame questions to invite open responses and within-group discussion. The second step, identifying the sample, drew on the 16 intervention classes, from each of which a focus group of six students was formed. This offered homogeneity in age, class teacher and experience of the teaching following the teachers' writing residential. The students were selected by the class teacher, stratified by gender and by writing attainment, using national assessment data: this added some heterogeneity to the sample, ensuring greater representativeness of students from each class. In line with Flores and Alonso (1995), we feel this created a “*balance between the components of uniformity and diversity, achieving groups homogeneous in those characteristics that affect the discussed topic and groups that are heterogeneous in features that are not relevant in relation to it”* (p. 89). The interviews were conducted by one of the research team of six (Step 3): each researcher was allocated specific schools throughout the project and managed all project liaison, observed lessons, and built relationships with both the students and the teacher. This mitigated the power relationship between interviewer and participants, and all the interviewers were experienced in interviewing children. Each focus group was interviewed twice: firstly, immediately after the teachers returned from the residential, and 3 months later, after the intervention was complete. The interviews were audio-recorded and subsequently professionally transcribed. Steps 4 and 5 of the NFER focus group method are reported further below.

The analysis of the interviews was principally inductive, following a systematic process of thematic analysis outlined by Braun and Clarke (2006). This means that themes are “*strongly linked to the data themselves…without trying to fit it into a pre-existing coding frame, or the researcher's analytic preconceptions*” (p. 83). The coding was undertaken by the authors and the first step involved shared reading and initial discussion of the interviews. Then each coder independently coded the same interview and allocated an appropriate descriptive code to the data segments. This initial coding was shared, discussed and refined, then developed iteratively, with constant refinement of code labels and checking of appropriate attribution of data segments to codes as more interviews were coded. These were then clustered into sub-themes of related codes, and finally each sub-theme was grouped thematically under the constructs structuring the interview schedule. Throughout the coding process, the coders met to ensure consistency, particularly through constant comparison, which involved refining the codes, identifying their properties, and exploring their inter-relationships (Taylor and Bogdan, 1984, p. 126). When all coding and clustering was complete, a final check of all data segments in each sub-theme was made to ensure consistent application of coding agreements. The final set of themes and sub-themes is outlined in Table 1.

**Table 1:** The final set of themes and sub-themes derived from the analysis of the focus group interviews.

**Themes**	**Sub-themes**	**Definition**
*Perceptions of writing*	Genres and their characteristics	Types of writing and their characteristic features
		Positive characteristics	Features or qualities that make writing “good”
		Negative characteristics	Features or qualities that detract from “good” writing
*Perceptions of writers*	Writers' attributes	Personal characteristics that writers need/possess
		Writers' skills	Skills that writers need or use
*Perceptions about the Teaching of Writing*	Helpful for writing	Ways in which anything/anyone helps with writing
		Unhelpful for writing	Ways in which anything/anyone is unhelpful
		Perceived changes in teaching	New approaches/recent changes in teaching of writing
		Perceived impact of writer	Professional writers' impact on teaching and learning
*Perceptions of writing process*	Preparing for writing	Pre-writing activities; personal preferences/dislikes
		Drafting	Processes/strategies involved in drafting a text
		Improving writing	Processes/strategies involved in improving a draft
*Enjoyment and motivation*	Enhancers	Factors that enhance motivation/enjoyment of writing
		Detractors	Factors that reduce enjoyment/motivation
		Personal writing outside school	Forms of writing that students engage in for pleasure
*Confidence and perceived skills as writers*	Self-description	Labels students use to describe themselves as writers
		Perceived strengths	Personal strengths or capabilities as writers
		Perceived weaknesses	Personal weaknesses as writers
		Perceived progress	Aspects of writing improved
		Helpful for confidence	Factors that enhance self-confidence
		Unhelpful for confidence	Factors that undermine self-confidence

Trustworthiness in qualitative research refers to the confidence of readers that a research study has been conducted and reported in a rigorous manner. It is not concerned with replicability as in quantitative research, it is concerned with trust and transparency. Table 2 provides a summary of trustworthiness in this study.

**Table 2:** The trustworthiness of the study.

Credibility	•Theoretical triangulation- approaching the topic from both cognitive and sociocultural perspectives • Careful reporting of multiple perspectives in the focus groups, including minority or outlier perspectives • Use of two interviews per focus group to accurately identify changed perceptions, allowing member-checking
Transferability	•Sampling strategy balanced by school type, gender and writing attainment to maximize representativeness of the data • Providing a thick description of the data • Constant comparison throughout the coding process
Dependability	•Systematic use of thematic analysis for data coding • Detailed reporting of focus group interview methodology to allow replication
Confirmability	•More than one coder involved in the coding process • Iterative development of a clear coding scheme

## 4. The outcomes of the focus group interview analysis

In order to address the research question (*How do students respond to a changed classroom environment for writing, and how does this connect with their motivation to write?)* we will draw on three of the themes—perceptions about the teaching of writing; enjoyment and motivation; and confidence and perceived skills as writers—as these evidence most closely the students' responses to their changed classroom environment for writing and their motivation to write. In presenting the data below, all quotations from the interviews are in italics with speech marks, and we indicate in brackets whether a student was in our primary school sample (aged 7–11) or in our secondary school sample (aged 12–14). The quotations used have been selected to exemplify both typical responses in a theme, and also the diversity of responses.

### 4.1. Perceptions about the teaching of writing

This theme clustered together comments where students expressed their views about how they were being taught writing, and strategies and approaches which they felt benefitted or hindered their learning as writers. This theme reflects particularly the teaching practices which characterized the classroom environment, and provides a context for later themes, as the students do talk explicitly about changed pedagogical practices which they found helpful. Four sub-themes were generated within this theme, as detailed in Table 3.

**Table 3:** The sub-themes of the perceptions about the teaching of writing theme.

**Sub-theme**	**Definition**	**Exemplar data segments**
Helpful for writing	Teaching strategies or behaviors which help with learning about writing	“She talks us through what we could improve on” [Freewriting] “*it helps you get your ideas flowing*”
Unhelpful for writing	Teaching strategies or behaviors which are unhelpful with learning about writing	[Prescribed topics] “*I don't know how to write it if it's not my story”*
Perceived changes in teaching	New or changed teaching strategies or behaviors noticed since the writing residential	“*it's less kind of about rules and it's more about creativity”* “*before we were like locked up and we had to do stuff we were told to do, now we've been let out”*
Perceived impact of writer	Ways in which the professional writer has impacted on teaching and learning about writing	[They give] “*a professional view on writing…like how she plans and how she writes it down and how she sees the work”* “*The writer told us how to zoom in on our stories and make them better…like put all the detail in”*

Students identified a range of strategies in their lessons which they found **helpful for writing**, and which were largely common across both pre- and post-intervention interviews and across all focus groups. These included, for example, teacher scaffolding and modeling of writing; sharing of ideas for writing; the use of exemplar materials and reference resources; one-to-one support when stuck with writing; and teacher feedback and target setting. These reflect typical national practices in the teaching of writing in England at the time, shaped by curriculum guidance and national assessment. However, some aspects were much more prominent in the post-intervention interviews and some new features emerged. For example, there was an increased emphasis on the value of individual support and feedback from both teachers and peers, particularly in relation to editing. Sharing ideas and writing as a class or with partners was also more frequently cited, especially as a resource for “magpie-ing” (borrowing ideas or language choices from others). This included noting the benefit of teachers who wrote alongside and shared their writing, which meant “*we can sometimes use some of the ideas from hers”* (Primary). There were new references to freewriting and its affordances, which suggest that students found it eased the problems of starting writing. For some, freewriting appeared to reduce the cognitive load of attending simultaneously to idea generation and accurate transcription: “*it's helpful because while you're writing, it doesn't make you think like, oh, I've got to do punctuation, I've got to do this and that. You can just do it after”* (Secondary). For others, it was principally a strategy which enabled idea generation and imaginative engagement: “*it helps you get your ideas flowing”* (Secondary); and “*it helps us use our creative thinking…it lets you access your imagination more”* (Secondary). The use of artifacts and different environments for writing were also identified as helpful for idea generation and descriptive writing, because, as one student explained, being outside “*helps trigger ideas, whereas before just sitting in the classroom with loads of people talking and things, it was quite hard to think of anything” (Primary)*.

Students made far fewer comments about what they felt was **unhelpful for writing**—a total of just 13 comments across both interviews, compared with 264 comments on what they found helpful. This could be attributable to the absence of a direct question in the interview addressing this, and certainly in future research, it would be useful to explore this more explicitly. What students did comment on linked very directly with common teaching practices in England, which are sometimes very directional and over-scaffolded (Barrs, 2019). Students identified tightly-prescribed tasks or processes (such as obligatory planning) as inhibitors. Sentence starters, paragraph starters or “tight themes” were often perceived as disabling, with one student observing that “*I don't know how to write it if it's not my story”* (Secondary).

The post-intervention interview included specific questions about any **changes in teaching** they had observed since the writing residential. Students in all focus groups observed recent changes in teaching followed teacher attendance at the writing residential, and the majority found the changed practices supportive. A common theme was the perceived relaxation of pressure and prescription:

□ “*(she's) taking the pressure off us*” (Primary);
□ “*she's been a lot more open…(less) precise about what we're going to do”* (Primary);
□ “*it's less kind of about rules and it's more about creativity”* (Secondary);
□ “*before we were like locked up and we had to do stuff we were told to do, now we've been let out”* (Secondary).

Freewriting was again cited, this time as a newly-encountered strategy which offered particular creative license because “*you don't really have any limitations, so it's literally whatever you want it to be… basically you're free to determine your outcome* (Secondary)*.”* Students also noticed “more active” and “interactive” approaches to writing and greater collaboration, identifying talk partners, editing buddies, paired writing and peer feedback as supportive. They also appreciated more time and “*space to think”* and “*more space to just write,”* without having to “*worry about trying to get bits done right then and there”* (Secondary). Some students observed that teaching had become less didactic: their teachers had “*backed off a bit”* and were less inclined to “*spoon-feed”* (Secondary). Rather than provide detailed guidance, these teachers tended to offer “clues” or prompts, and encourage independent thinking:

□ “*(Before), she just gave us something to write down and we just wrote it. And now it's kind of thinking of our own ideas”* (Primary);
□ “*Before…she'd have like specific tasks, whereas now she just gives us an idea and we have to use our brains more* (Secondary)”*;*
□ “*I think she's helping us more by not helping us as much”* (Secondary).
□ “*(The teacher) is now a last resort for us”* (Secondary).

These comments regarding a less didactic approach were paralleled by observations that where teachers positioned themselves as co-writers, writing in the classroom, students were more aware of shared learning—“*She can learn at the same time, but then she can teach us what she's learned”* (Primary). This included recognition that the teacher as a writer does not know “*what they're doing 100% all the time … they don't really know what they're doing at some points”* (Primary).

In the post-intervention interviews, students also discussed the experience of being taught by **professional writers**. They welcomed writers' specialist expertise: they could provide “*a professional view on writing…like how she plans and how she writes it down and how she sees the work”* (Secondary), and “*you trust them”* (Secondary). Students noted writers' expert subject knowledge about “*good ways to do it”* (Primary), “*what works well”* (Secondary) and the “*qualities people look for”* (Secondary). This included comments on and growing purposefulness in editing through being advised “*to zoom in on our stories and make them better…like put all the detail in” (Primary)* or “*how to cut in, like cropping a picture but you like cut into what you're actually supposed to be writing about, other than like trailing off”* (Primary).

Writers' approaches to teaching were widely regarded as both “fun” and “helpful*.”* In particular, students identified their help with idea generation: “*the way (the writer) teaches it helps you a bit more because he knows how difficult it is to think of the ideas”* (Secondary). Their use of “fun scenarios*,”* “stories*,”* modeled examples and suggested possibilities “*makes your imagination run wild”* (Secondary). Writers were perceived as encouraging—“*by saying you can do it”* (Primary) and as promoting a sense of ownership—“*he said it doesn't mean that ours is wrong*—*it's just the way we think of it”* (Primary); “*it doesn't matter what other people think, it's about what you think”* (Primary). They also provided “inspiring” role models. According to one primary group, having a writer in the classroom: “*gives you ideas”; “gives you a voice”; “gives you an idea of what you want to do when you're older”; “(gives you) a sense of what you need to do (to be a writer)”; “helps us improve stuff”; “sharpens the mind”* and “*inspires us.”*

However, not all students welcomed all aspects of change in teaching. Some found freewriting stressful and preferred more structure and time to plan: “*I like having a subject to write about more than making up something, because I find it hard* (Primary)*.”* Peer feedback was not always regarded as helpful, often because it was insufficiently critical, and some students preferred to rely on their own judgement. A few students found professional writers “*just a bit over the top”* and “*intimidating”*: “*having a professional writer, you feel like, oh, this has got to be perfect, and if I read it out and it's not good…he's going to criticize me”* (Secondary).

In summary, students' perceptions of the teaching of writing show awareness of the teachers' changed classroom practices after the intervention. In particular, many students enjoyed freewriting because of the freedom it gave them and its support for ideation. They also noted the less didactic teaching with reduced teacher control of the writing tasks and greater student freedom, and they welcomed the expertise of the professional writers. Some students, however, found the greater freedom and reduced teacher direction less helpful.

### 4.2. Enjoyment and motivation to write

This theme focuses more directly on students' reported enjoyment of their writing lessons, and the established positive connection between enjoyment and motivation to write (Reeve, 1989; Zumbrunn et al., 2019). The pre-intervention interviews invited students to consider whether they enjoyed writing or not, and whether they felt pleased with their writing. This was followed up in the post-intervention interviews with more focused questions on their enjoyment of the writing they had been undertaking during the intervention. The sub-themes are presented in Table 4.

**Table 4:** The sub-themes of the enjoyment and motivation to write theme.

**Sub-theme**	**Definition**	**Exemplar data segments**
Enhancers	Aspects of the teaching of learning and writing which enhanced enjoyment and motivation	“*The thing I most enjoy about writing is how much you can use your imagination …it really is just something of your mind that will go the reader and say ‘wow'!”*
Detractors	Aspects of the teaching of learning and writing which detracted from enjoyment and motivation	“*I actually hate planning for creative writing… it kind of stops the freedom because if you're sat there planning, say, a mind map…it's just really irritating, especially if you already have the entire story plot in your mind”*

Across both sets of interviews and all age groups, **enhancers** of enjoyment related to creative freedom, autonomy and ownership, and use of imagination—typical comments included:

□ “*I just like being free when I write…I like being in my head when I'm writing. I like writing what I'm thinking, what I like. I just enjoy writing…whatever I want”* (Primary);
□ “*The thing I most enjoy about writing is how much you can use your imagination…it really is just something of your mind that will go the reader and say ‘wow'!”* (Primary);
□ “*I really, really do like creative writing because I think that I can just kind of like set my mind kind of like free, just like let everything out because it's my piece of writing and like, well obviously, you can criticize it but it's my point of view”* (Secondary).

With few exceptions, students favored creative genres such as story writing, poetry, and play scripts, which they associated with greater freedom and personal expression. Approximately one quarter of students claimed to write at home for pleasure either regularly, “*occasionally”* or “*when bored,”* and invariably chose creative forms. Aside from text messaging, Facebook and snapchat (which they didn't count as “*long”* writing), the most frequently cited genres were stories, poems, songs and diaries.

In contrast, **detractors** from enjoyment included non-fiction writing, notably report writing and essays, which they associated with rules and constraints, such as having to use “PEE” paragraphs (a widely-used paragraphing scaffold in England: Point; Evidence; Example). They also disliked prescribed tasks and topics:

□ “*I don't like the fact that most of the time you just have…to do stuff that the teacher says”* (Primary);
□ “*I don't like people telling me ‘you have to write this”'* (Primary).
□ “*I don't like doing things I'm told to do”* (Secondary).

Some students found inflexible routines such as planning before writing or correcting after writing painful and demotivating:

□ “*I actually hate planning for creative writing…it kind of stops the freedom because if you're sat there planning, say, a mind map…it's just really irritating, especially if you already have the entire story plot in your mind”* (Secondary);
□ “*I hated it when I went to [the teacher], because then my dream was just crushed…it's taken me at least two days now to complete two words…she put all the mistakes in and I have to go and correct it now, and it's killing me”* (Primary).

One clear message in the post-intervention interviews was the enjoyment students derived from new approaches to classroom writing. Whilst this might be due to a sense that they were supposed to enjoy the post-intervention teaching, the detail in the comments show precise reflections on what enhanced their enjoyment, and did relate specifically to teaching strategies encouraged in the intervention. Many of the students described the changes in teaching approach as liberating and “*fun,”* in terms of pleasure: “*It's a lot more free and it's not as strict, because we get to kind of relax and just have fun and just write”* (Secondary); and in terms of excitement: “*(writing before) was pretty boring, but this is more exciting…I like it when I can write without having to think about it”* (Secondary). They identified increased freedom to create and exercise their imaginations as significant, and freewriting was enjoyed particularly.

Students welcomed the more flexible approaches to generating writing, not only the freewriting which “*really let my ideas flow”* (Secondary) but also the greater attention to drafts: “*I've enjoyed doing drafts, because before we didn't do drafts and it's a lot harder to edit it and find every detail. But when you look through it and then you make another draft it's a lot easier” (*Primary). They also enjoyed a greater emphasis on interactive and collaborative approaches, including more sharing of ideas, talk partners, and peer review: “*The whole classroom has become more relaxed—you can share ideas and feedback* (Secondary*).”* Some students identified more relaxed classroom environments for writing (e.g., shoes off; teacher as co-writer) and different locations for writing as promoting engagement.

The encouragement of teachers and professional writers were felt to impact positively on motivation:

□ “*(the writer) was well enthusiastic…it makes you want to do it more” (*Secondary);
□ “*you want to put your best into it…you want to make an impression to show that you're capable of the same level when you get older”* (Secondary);
□ “*if she likes something in your book she tells you to do it at home and like she encourages you to do more writing at home”* (Secondary).

Some also felt their attitude to writing had changed or that they were more inclined to engage in writing beyond school as a consequence of the intervention:

□ *In September I didn't really like writing, now I do* (Primary);
□ *I didn't really like writing but now I'm getting into it. I can do more* (Secondary);
□ *At the start I didn't really enjoy poetry as much, and when (the writer) came up with the “I remember” poem idea, I've written stuff at home and I've used that kind of technique* (Secondary).

Some students observed changes in behavior and motivation post-intervention, with one student observing that “*People mucking about has gone down—they enjoy the tasks more so they're putting more into it”* (Secondary).

However, enjoyment of intervention activities was not unqualified. A few students disliked the more open writing briefs, preferring a structured approach and greater guidance. Freewriting was sometimes perceived as too pressured:

□ “*These past few weeks have been rushed writing. I like to plan and just pause and think… setting what it should be, the structure, knowing what to do”* (Secondary).
□ “*The ‘just write' thing is freeing in some ways but also it pins you down because you know you have to produce a good piece within five minutes of thinking of it…often I don't have ideas straight away”* (Secondary).

For some, the emphasis on editing was tedious and stressful: “*(it) takes loads of time”* and “*makes you stress, you just want to feel free and do what you want in your stories”* (Primary). Less confident writers did not enjoy sharing their writing aloud, because “*I feel like I just can't compete”* (Secondary) or because of dissatisfaction with their writing, “*I always think I can do something better”* (Secondary). Others disliked peer review and found unhelpful feedback from peers irritating, for example, “*you ignore it because sometimes they just point out all your missing full stops and that's it”* (Secondary).

Overall, the students' responses in this theme demonstrate a strong link for many between enjoyment of writing and motivation to write. Greater creative freedom was associated with increased agency and ownership of writing, and greater emotional engagement, whilst the provision of supportive feedback, including peer feedback was welcomed.

### 4.3. Confidence and perceived skills

The pre- and post-intervention interviews sought to elicit students' confidence in themselves as writers, and their own self-efficacy perceptions, in order to determine whether the intervention had in anyway altered their self-perceptions. The analysis generated six sub-themes, as described in Table 5.

**Table 5:** The sub-themes of the confidence and perceived skills as a writer theme.

**Sub-theme**	**Definition**	**Exemplar data segments**
Self-description	Labels students use to describe themselves as writers	•“*sometimes good, sometimes bad”* • “*I don't know, it kind of depends on whether anybody else says it's good or not”*
Perceived strengths	Personal strengths or capabilities as writers	•“*my imagination”* • “*I don't make many mistakes in my writing”*
Perceived weaknesses	Personal weaknesses as writers	•“*I struggle to get started”* • “*After a while I just get a bit distracted”*
Perceived progress	Aspects of writing improved	Finding “*it easier to think of things to write”* • “*before I didn't use as much description as I do now”*
Helpful for confidence	Factors that enhance self-confidence	“*Because we've had the chance to write what we want…it makes you more proud of what you've done because it's more yours”*
Unhelpful for confidence	Factors that undermine self-confidence	[Sharing writing] “*When my friends read it out, I'm like oh I wish I could be like that. They're much more better than me, so I put myself down”*

In the pre-intervention interviews, the student **self-descriptions** of themselves as writers divided rather evenly across different proficiency levels. Approximately one third described themselves as “*good”* writers who were usually pleased with the writing they produced. A further third claimed they were “*OK*” writers or were “*sometimes good, sometimes bad*,” often depending on the nature of the task or their interest in the topic. A final third considered themselves “*not good”* or “*rubbish”* and a number felt they couldn't judge: “*I don't know, it kind of depends on whether anybody else says it's good or not”* (Secondary). The majority of students also felt their writing had improved gradually over time, with their **perceived strengths** being accuracy, use of the imagination, and ideas for writing. Nevertheless, when reflecting on **perceived weaknesses**, half of all responses across the age groups described difficulties with the “*struggle to get started”* (Primary) and with idea generation. These comments indicated a sense of personal inadequacy in this area: the inability to “*think of ideas”* (Primary), “*to come up with ideas”* (Primary), and for one student, the perception that “*I just don't have any ideas”* (Secondary). A small number identified weaknesses in vocabulary and the need to “*look in a thesaurus more often” (*Primary) and problems with concentration on the writing task, with one student reflecting that “*After a while I just get a bit distracted”* (Secondary).

In the post-intervention interviews, the students made fewer comments about their perceived self-efficacy, but made significantly more comments about their perceived progress, talking about their improvement in relation to the teacher intervention rather than the more general comments about progress over time which featured in the pre-intervention interviews. They also made more comments post-intervention about what supported or diminished their confidence, albeit these numbers are small (see Table 6).

**Table 6:** Showing the number of data segments coded to each sub-theme.

**Sub-theme**	**Pre-intervention**	**Post-intervention**
Self-description	66	47
Perceived strengths	58	52
Perceived weaknesses	69	40
Perceived progress	40	126
Helpful for confidence	4	20
Unhelpful for confidence	4	11

Some of the comments on **perceived progress** cited general improvements: for example, one student maintained that “o*ver, say, the last six, seven weeks I've improved drastically…it's had a massive impact”* (Secondary) whilst another felt “*It's made my levels go higher”* (Secondary). However, many of the comments referred directly to the changed teaching strategies introduced after the writing residential, including the use of personal notebooks—“*the orange books* [personal notebook] *and all the new ways we're being helped have definitely helped me a lot”* (Primary). There was particular reference to improved ability to generate good ideas, with students reporting finding “*it easier to think of things to write”* (Secondary), being “*better at making stuff up”* (Primar*y)*, and perceiving that “*the teacher likes my stories more now…I've got better ideas”* (Primary). At the same time, far fewer students identified generating ideas as a **perceived weakness**. Other perceived improvements included increased accuracy and control in text structure: “*I've definitely improved on ending and beginning sentences, paragraphs, punctuation, lots of things…all because of (the teacher) and the support she's given us in our story writing”* (Primary); the use of greater descriptive detail (“*before I didn't use as much description as I do now”);* and vocabulary choice, using “*a wider range of vocabulary”* (Secondary).

The students reported high levels of satisfaction with the writing they had produced over the course of the project, with many claiming their confidence had improved:

□ “*I've got more confident with my writing”* (Secondary*);*
□ “*Because we've had the chance to write what we want…it makes you more proud of what you've done because it's more yours”* (Secondary);
□ “*Usually I'm not confident…because I don't think I'm very good…but because of this little project I feel a bit more confident with my work because occasionally it's actually quite good and it makes sense”* (Secondary);
□ “*I feel a bit more free with my writing…like I feel I could write more…I feel more confident when I'm writing stuff like this”* (Secondary).

Students often associated progress and increased confidence with perceived shifts in classroom approach—in particular, the greater emphasis on idea generation; the use of new drafting and revising strategies which had made writing easier; and more opportunities for collaboration and feedback at formative stages of writing. Improvements in idea generation were often attributed to “*warming up”* activities and sharing of ideas before writing, which “*helps my mind get going and I'm writing better stuff”* (Secondary). Writing activities which encouraged students to draw on their personal experience or memories as a basis for narrative were perceived by some to have strengthened confidence because “*starting with the memories and then making it more imaginary…gave us more confidence in a way”* (Secondary). They were also viewed as increasing the sense of ownership and individuality: “*it's highly unlikely as well that someone's going to have written the same sort of story as yours, which is a nice feeling that you've written your own work”* (Secondary). The emphasis in project activities, and particular the writers' testimonies, on the value personal experience or observation offered for story-writing appeared to have altered some students' self-regulation of their writing strategies:

□ “*Well [the writer] said he got his inspiration from real life, like, occurrences, like road names, he'd use them…I've started to use my real-life experiences of seeing things and put that in to a story which I'm going to write for my assessment”* (Secondary);
□ “*I have used life experiences and all those things, because you can't just make them out of nowhere. But I'm, sort of, looking out for them now”* (Secondary).

Students' perceived progress was also linked to improvement in revising/editing skills as a consequence of changed teaching strategies. Younger students claimed to have started editing or were doing “more editing*.”* This involved re-reading for sense, correcting errors, improving word choice and sometimes more substantive change such as restructuring for reader impact:

“*I read through my story and mine features a ghost. And in the second paragraph it already started about who it was. And then I read through it and actually they could, if I got rid of that bit and put it at the end…then the readers could guess who it is”* (Primary).

Some older students described revising their texts in new ways. For some, this related to being more inclined to re-read and “*check*” their work, noting for example, a change from limited attention to revision where “*occasionally I've changed a few sentences…but half the time I didn't”* to greater awareness of the value of re-reading—“*now I will always read, I'll try and get through all of it”* (Secondary). Other students described paying greater attention to deleting unnecessary material and making every sentence count:

□ “*In the past I used to…just throw everything in there, you know, ramble. But now I think about every sentence I write and if I feel it doesn't fit…I do cross it out*—*that makes for very messy writing!”* (Secondary);
□ “*I used to ramble quite a lot. And now I think about every single sentence I write, like it has to be part of the story…so I'll write a draft, and then I'll think what I don't need…it may work but it's not relevant to the actual story, it doesn't need to be in there.”* (Secondary).

A smaller number of students identified which teaching strategies they perceived as **helpful for confidence**. New drafting strategies such as freewriting were perceived to have helped with fluent idea generation and facilitated a more effective writing process, where planning was conceived more broadly than an outline of the intended text. One student reflected that when “*we have to like plan out our things, sometimes I do the freewriting thing just so I can put it down and it's kind of a draft in itself”* (Secondary). Another student, referring to the experience of being co-taught by the professional writers, had learned about a more flexible approach to generating ideas, where “*instead of just putting like one idea and just sticking with it, you can put multiple ideas and then choose whatever one you want, and edit it”* (Secondary). Students also noted the more collaborative writing environments, which included sharing of writing, as helpful to “*build confidence.”* For some, this way of working was “*less competitive”* (Secondary). Younger students in particular seemed to find support in the mutuality of collaborative working because “*I help her with her writing and she helps me with mine”* (Primary); and “*if you have a problem you can just ask (your talk partner) and they can help you”* (Primary). The collaborative writing environment also built confidence through creating space for positive or “*constructive”* feedback—“*we read all our homework out and she said just give positive feedback and it helped and made you feel nice about what you've done”* (Secondary). When teachers shared their own writing problems or got emotional reading their work aloud, students identified with them and felt reassured:

□ “*People think, ah, she's an English teacher, she should be confident, proud in her work, but she's not, she has insecurities about her work and obviously we can relate to her”* (Secondary*);*
□ “*When she was reading it, she started turning round because she got emotional… don't be scared when you're reading your work out”* (Primary).

However, for a few students the changed teaching strategies were perceived as **unhelpful for confidence**. Those already less confident writers sometimes found hearing their friends reading their work demoralizing because, as one student expressed it, “*oh I wish I could be like that. They're much more better than me, so I put myself down”* (Secondary). For these students, sharing their writing was a fearful experience, making them “*so scared that other people would judge it badly.”* Equally, in contrast to the many student observations of the helpfulness of freewriting, a minority found it difficult and felt they had lost confidence or that their writing had deteriorated: “*I think I'm going the opposite way with my creative writing…when I was little, I used to be really creative, but now it's kind of just going”* (Secondary).

Comments in this theme show many students were more aware of perceived progress in writing post-intervention than increased self-efficacy, and increased confidence attributed to a more collaborative environment, sharing work with each other.

Overall, the analysis of the focus group interviews shows clear recognition of the changed practices in teaching writing during the intervention, and in general, the students responded with positivity and enjoyment to these changes. Whilst there is always the possibility of a halo effect in their responses, their references to specific strategies or practices with high alignment to those of the teachers' residential experiences suggests they are genuinely commenting on the particular changed writing environment encouraged by the Arvon writing community.

## 5. Discussion

Before discussing the implications of these findings for our understanding of motivation to write, it is important to be cautious, even parsimonious, in how we interpret these data. Firstly, they are highly context-specific. The Arvon writing residential is founded upon a very definite sense of values and commitment to a particular kind of writing community. The teachers in our study were willing to attend the residential despite its demands on their own free time, so may not be representative of all teachers of writing: certainly some were already keen writers, and others were motivated by a desire to learn more about being a writer in order to help their classroom practice. The writing undertaken was creative writing, thus not reflecting the wider range of writing types student are expected to master as they mature as writers. The focus group interviews are time-specific and closely linked to the intervention, and we cannot be sure that the student responses would be sustained over time. Secondly, the analysis synthesizes the data into themes and sub-themes across the dataset, but this is not generalisable, even within the dataset—for example, not all teachers gave the students notebooks for “messy” writing. As would be expected, the transfer of learning by the teachers from the Arvon writing residential was not uniform, and was mediated by the teachers in different ways. Therefore, in the discussion which follows, we seek to explore the implications of the students' responses through consideration of two over-arching themes, rather than focusing too closely on particular details, and from this suggest fruitful lines of enquiry for further research.

### 5.1. The importance of autonomy and choice in writing

One over-arching theme running through the students' responses is how they valued the greater autonomy and choice that they experienced in the post-residential intervention. This connects with the emphasis on autonomy and autonomous motivation in the research (Ryan and Deci, 2000, 2020; Turner et al., 2014; Robinson, 2023), and particularly with study De Smedt et al. (2020) in the context of reading and writing. The student comments indicate how they value being able to exercise volition when learning to write: they referred especially to freewriting, which supported idea generation and allowed them to follow their own ideas. They also enjoyed having writing notebooks, or “messy” books, which again gave freedom about what to write, but also freedom for teacher intervention and evaluation. For some, the new sense of autonomy was expressed in terms of greater ownership of their work *(“my story”; “my point of view”*), and a reduced dependence on the teacher, who for one student had become a “*last resort.”*

In parallel to the students' espousal of autonomy in writing as a positive thing, their dislike of being controlled was also evident, particular when they talked about what diminished their enjoyment and motivation to write (the sub-theme, “detractors”). They dislike the teacher telling them what they have to write, having “*to do stuff that the teacher says,”* and having “*to stay in the boundaries.”* On one level, this relates to the desire for greater choice about topic and what to write, but it also relates to very constrained writing practices. The students noted changes in their teachers' behaviors, such as being less prescriptive about what they were doing, having less emphasis on rules, and less constraint—or as one student pithily expressed it, “*before we were like locked up and we had to do stuff we were told to do, now we've been let out.”* The students' reflections regarding a lack of autonomy in the writing classroom echo broader national concerns about a highly-constrained writing curriculum (Bearne, 2017; Barrs, 2019; Hardman and Bell, 2019). Typical writing practices in England involve a high level of direct instruction, tending to tell students exactly how they must write a particular text, and more oriented toward normative compliance than to fostering understanding of how texts work and how writers' choices can shape reader responses. Of course, teachers themselves do not have full autonomy in teaching writing according to their own interests and values as many are required to teach within the expectations of a specified writing curriculum, or with specific writing assessments in mind.

### 5.2. The importance of a collaborative writing community

The second over-arching theme emerging from our analysis is the students' recognition of a change in the atmosphere of the writing classroom. They felt that the classroom had become more relaxed, and less pressured, and one where collaboration was actively encouraged. The use of talk partners and writing buddies was received positively, and students seemed to enjoy the opportunity to “*share ideas and feedback.”* The sense of a collaborative writing community was strengthened by the visibility of the teachers as writers themselves, sharing their writing, but also sharing their vulnerability, such as becoming emotional when reading aloud their writing, and revealing “*insecurities about her work.”* It also involved teachers being positioned as learners within the community, who can “*learn at the same time”* as the students, and not necessarily be certain about everything. From a socio-cultural perspective, Prior has argued that “*teachers in schools are always co-authors (often dominant ones) in students writing”* (Prior, 2006, p. 58) because of their role in the production of student texts through determining what students, when they write, and the changes made through informal and formal feedback. However, what is perhaps more evident in the classrooms in our study is a sense of teachers as co-writers, not from a position of superiority, but from one of shared learning.

Both Hidi and Boscolo (2006) and Pajares and Valiante (2006) refer to collaborative writing as motivational, but the students did not mention collaborative writing, where one text is produced by two or more authors. What the students seem to be discerning is a change to a more collaborative community of practice for writing, bringing together people with a shared purpose, and engaging in “*a process of collective learning in a shared domain”* (Wenger-Trayner and Wenger-Trayner, 2015). This collective learning community may also have created a stronger sense of the meaningfulness of the writing. The notion of meaningfulness of a task or domain has been linked with motivation (Wigfield and Eccles, 2002; Behizadeh, 2014) and Hidi and Boscolo argue that such meaningfulness is less about the writing tasks themselves but is “*deeply rooted in the context in which writing is a meaningful authentic activity*” (2006, p. 144). The students made no direct reference to meaningfulness, but the comments in Table 6 above may indicate that the changed ways of working together, including the greater autonomy, allowed for more emotional engagement with the writing as intrinsically meaningful to them.

### 5.3. A motivational climate for writing

The two over-arching themes discussed above are less about specific teaching strategies than they are about the context in which writing occurs. They point to the importance of the environment for writing and how it can be a motivational climate for writing. Robinson (2023) argues that the motivational climate is not simply about observable teaching practices but about how students feel about their teaching and the meanings they create from it. Certainly, the responses of the students in our study reflect more than the like or dislike of particular teaching strategies. The students may have shown high appreciation of the freewriting strategy, but this might diminish over time if repeatedly used over time: its significance is in the autonomy it offers. Previous research on motivation has often identified constructs or characteristics which lead to higher motivation. For example, Turner et al. (2014) structured an intervention around the principles of autonomy, competence, belongingness and meaningfulness; Linnenbrink-Garcia et al. (2016) focused on the need to support students' feelings of competence, autonomy, use personally relevant and active tasks, emphasize learning and de-emphasize social comparison, and encourage feelings of belonging. Specifically related to writing motivation, Bruning and Horn (2000) synthesized research findings into four constructs: nurture positive beliefs about writing; establish authentic writing goals; generate a supportive context; create a positive emotional environment. However, it may be more valuable to think more specifically about a writing environment, within which these characteristics might be integrated, and to conduct more studies which look more holistically at the environment for writing.

As Camacho et al. (2021) review indicates, research in writing motivation has tended to focus predominantly on self-efficacy. Although there have been studies which have investigated the relationship between particular teaching strategies and motivation, these focus on the strategy not the teacher. However, it may be even more important to consider the role of the teacher in establishing a motivational writing environment. Research has addressed teacher competence or self-efficacy in teaching writing (Cutler and Graham, 2008; Hodges, 2015; Wright et al., 2019), rather than considering their identity as writers. The writing residential attended by the teachers changed, to varying degrees, their identity as writers and their stance toward writing: it was this change that translated into the way they altered the environment for writing. Further research might focus more on the relationship between a teacher's identity as a writer and how this plays out in the classroom environment. At the same time, it is important to take account of the realities of the classroom and the educational context. Whilst a writing community might ideally involve members being “*mutually engaged in using writing to accomplish a desired purpose”* (Graham, 2018, p. 259), in many writing classrooms there is limited mutual engagement, and teachers struggle to engage students in writing activities. Although a more constructive writing environment might enable better engagement and motivation to write, in practice, many teachers are juggling with externally-imposed constraints which may conflict with their own espoused beliefs and enacted practices.

## 6. Conclusion

In this paper, we have highlighted the importance of the classroom environment in supporting and sustaining motivation to write. In particular, we have pointed to students' positive responses to the collaborative environment they experienced, resonating with Bruning and Horn's advocacy of “*a climate of trust, caring, and mutual concern”* (Bruning and Horn, 2000, p. 34), and their valuing of autonomy and choice. These facets are strongly linked to the nature of the intervention, and further research in different contexts is needed to investigate this further. It is also important to investigate the balance between student autonomy and teacher control, particularly in relation to direct instruction. Given the known importance of explicit teaching of writing (Graham and Perin, 2007), it may be possible to conceive of writing environments where direct instruction is not perceived by students as synonymous with loss of autonomy.

Greater attention to the writing environment would also benefit from more integration of sociocultural and sociological perspectives on writing, which foreground writing as social practice. The Writer(s)-within-Community model (Graham, 2018) is significant in bringing together cognitive and socio-cultural insights, and in emphasizing the notion of a writing community. It conceptualizes the writing community as layers of contextual interactions, including the immediate community of writers, their purposes and collective histories, and also the broader contextual influences from policy, culture and history. Further inter-disciplinary research, ideally with researchers from different disciplines, might usefully expand on this by incorporating sociological thinking about identity, and about structure and agency into existing cognitive perspectives. This has implications for the design of future research, and particularly, writing interventions. Wigfield and Koenka (2020) have suggested that motivation research needs to take a new direction by moving away from interventions focused on individual student motivation toward interventions more attentive to the learning context. Echoing this, we would argue for a more situated perspective on motivation in writing which recognizes that motivation is not simply an internal characteristic of an individual but is situated within the context of a community of writers.

## Data availability statement

The raw data supporting the conclusions of this article will be made available by the authors, without undue reservation.

## Ethics statement

The studies involving human participants were reviewed and approved by the University of Exeter, United Kingdom and the Open University, United Kingdom. Written informed consent from the participants' legal guardian/next of kin was not required to participate in this study in accordance with the national legislation and the institutional requirements.

## Author contributions

DM and TC were responsible for the research design, project management, and data analysis. LO led on the data collection and analysis of the student interviews. DM wrote the manuscript. All authors contributed to the manuscript and approved the submitted version.
