# Supplementary material for: The impact of a changed writing environment on students' motivation to write
Source: Front Psychol. 2023 Oct 30;14:1212940. doi: 10.3389/fpsyg.2023.1212940 (PMC10642545; doi:10.3389/fpsyg.2023.1212940)
Supplement: Supplementary file 1 [file Data_Sheet_1.docx]

**APPENDIX A: DESCRIPTION OF THE INTERVENTION**

The intervention was a five day writing residential for teachers, led by creative writing charity, Arvon (https://www.arvon.org/). As part of its mission to promote creative writing, Arvon runs residential writing courses for different groups, including teachers and students. Their courses are founded upon four value statements which shape the writing environment participants’ experience during a residential:

INCLUSIVE: Everyone is creative. We make spaces that are open to all, where anyone, regardless of writing experience, feels welcome and included as part of a community of writers.

INSPIRING: Step away from the routine, be inspired by writers and our beautiful places and release your imaginative potential. Arvon is a place for contemplation, challenge and going beyond what you thought you were capable of achieving.

SUPPORTIVE: Creative writing is a craft that can be learnt, through guidance from experts, and through the peer support that comes from creative friendships with fellow writers. At Arvon, writers teach writers, and everyone encourages each other to become a better writer.

TRANSFORMATIVE: Immersing yourself in creative writing nourishes the imagination, can deepen the connection to self and to the world, and can lead to dramatic change and progress.

https://www.arvon.org/about/arvon-home-of-creative-writing/

**THE TEACHERS’ RESIDENTIAL PROGRAMME**

The residential was led by two professional writers who took responsibility for planning and preparing the programme, and for establishing the sense of a community of writers amongst the teacher participants.

|  | **Morning** | **Afternoon** | **Evening** |
| --- | --- | --- | --- |
| Day 1 |  | Arrival and welcome | Workshop 1 hour |
| Day 2 | Workshop 2.5 hours | Individual Tutorials 0.5 hours  Time and Space for writing | Tutor 1: Reading of own writing |
| Day 3 | Workshop 2.5 hours | Individual Tutorials 0.5 hours  Time and Space for writing | Guest Writer reading of own work |
| Day 4 | Workshop 1.5 hours  Professional Development | Individual Tutorials 0.5 hours  Time and Space for writing | Tutor 2: Reading of own writing |
| Day 5 | Workshop 2.5 hours | Teachers preparing together an anthology of work produced during the residential | Teachers sharing reading of their work in the anthology |
| Day 6 | Departure |  |  |

**Description of Residential Activities**

Workshop:

The writing workshop involved a substantial amount of freewriting to generate ideas, initiated by a range of different prompts – such as shared reading of a poem; artefacts; or pictures. Participants shared their writing work-in-progress during the workshop.

Individual Tutorials:

Each teacher had two one-to-one tutorials during the week, one with each of the professional writers. In the tutorials they discussed their writing experiences and shared and received focused critical feedback from the tutor.

Professional Development:

This session, towards the end of the residential, created an opportunity for the teachers to reflect on what they had learned during the residential about being a writer and teaching writing, and what they might change in their own professional practice.

Time and Space for Writing:

This was free time for the teachers to write, reflect on ideas, or to have a break from writing, making use of the natural environment of the residential centre.

**APPENDIX B: FOCUS GROUP INTERVIEW SCHEDULES**

**Theoretical constructs**:   Perceptions of writing; Perceptions of teaching; Understanding of writing process; Enjoyment/motivation; Confidence and perceived skills

| **INTERVIEW 1** | **INTERVIEW 2** |
| --- | --- |
| **Perceptions of writing** | **Perceptions of writing** |
| - Tell me about the kinds of writing you have been doing in English/Literacy lessons recently. - What do you think makes writing ‘good’? If you had to make a list what would you include? - Do you think anyone can be a good writer? Why/Why not? | - Tell me about the kinds of writing you’ve been doing in English/Literacy lessons since we last spoke. - What do you think you need to do to make this kind of writing ‘good’? *(probe for anything new they may have learnt since previous responses)* |
| **Perceptions of teaching** | **Perceptions of teaching** |
| - What sort of things does your teacher do to help you with writing? | - How has your teacher been helping you with this kind of writing? Have you noticed anything new that your teacher has done to help you? - Has having a writer in the classroom helped you? How? - Has anything or anyone else helped you become a better writer since we last spoke? |
| **Understanding of writing process** | **Understanding of writing process** |
| - What do you normally do to get ready for a piece of writing? *(E.g. draw; read/listen to examples; brainstorm; talk; make a plan)* - What do you do if you get stuck with writing? *(E.g. reread so far; look at examples; talk to a friend; ask the teacher)* - How do you go about improving a piece of writing?  *(E.g. reread and make changes; use feedback from peers/teachers; check for errors; redraft)* - What kind of changes do you usually make? *(Corrections; vocabulary; additions/deletions; structure)* | - What have you been doing to prepare for writing? Have you tried any new ways of getting ready for writing? - What sort of things have you been doing to improve what you’ve written? Any new things? - What kind of changes have you been making to improve your writing (if any)? |
| **Enjoyment/motivation** | **Enjoyment/motivation** |
| - Do you enjoy writing? What do you enjoy most? (Your answers might vary depending on the type of writing) - Are you usually pleased with what you’ve written? Why/why not? - Do you write in your own spare time? What kinds of writing? *(E.g. diaries; stories; poems; lyrics; social media; letters)* | - Have you enjoyed the writing you’ve been doing? What have you enjoyed most/least? - Are you pleased with what you’ve written? Why/why not? - Have you been doing any writing in your own spare time? What kinds of writing? |
| **Confidence and perceived skills** | **Confidence and perceived skills** |
| - Do you think you’re a good writer or not? In what ways (strengths and weaknesses)? - Do you think you’re getting better as a writer? In what ways? | - Do you think you’re good at this kind of writing? What do you think you’re good at/less good at? - Do you think you’re getting better as a writer? In what ways? |
